# A Sensitivity Analysis of an Inverted Pendulum Balance Control Model

**DOI:** 10.3389/fncom.2017.00099

**Published:** 2017-10-27

**Authors:** Jantsje H. Pasma, Tjitske A. Boonstra, Joost van Kordelaar, Vasiliki V. Spyropoulou, Alfred C. Schouten

**Affiliations:** ^1^Department of Biomechanical Engineering, Delft University of Technology, Delft, Netherlands; ^2^Department of Biomechanical Engineering, Institute for Biomedical Technology and Technical Medicine (MIRA), University of Twente, Enschede, Netherlands

**Keywords:** balance control model, human balance control, parameters, sensitivity analysis, frequency domain

## Abstract

Balance control models are used to describe balance behavior in health and disease. We identified the unique contribution and relative importance of each parameter of a commonly used balance control model, the Independent Channel (IC) model, to identify which parameters are crucial to describe balance behavior. The balance behavior was expressed by transfer functions (TFs), representing the relationship between sensory perturbations and body sway as a function of frequency, in terms of amplitude (i.e., magnitude) and timing (i.e., phase). The model included an inverted pendulum controlled by a neuromuscular system, described by several parameters. Local sensitivity of each parameter was determined for both the magnitude and phase using partial derivatives. Both the intrinsic stiffness and proportional gain shape the magnitude at low frequencies (0.1–1 Hz). The derivative gain shapes the peak and slope of the magnitude between 0.5 and 0.9 Hz. The sensory weight influences the overall magnitude, and does not have any effect on the phase. The effect of the time delay becomes apparent in the phase above 0.6 Hz. The force feedback parameters and intrinsic stiffness have a small effect compared with the other parameters. All parameters shape the TF magnitude and phase and therefore play a role in the balance behavior. The sensory weight, time delay, derivative gain, and the proportional gain have a unique effect on the TFs, while the force feedback parameters and intrinsic stiffness contribute less. More insight in the unique contribution and relative importance of all parameters shows which parameters are crucial and critical to identify underlying differences in balance behavior between different patient groups.

## Introduction

During human stance, the central nervous system (CNS) continuously has to compensate for deviations from an upright body orientation and the pull of gravity. The CNS estimates the body orientation based on sensory information from vision, the graviceptive (i.e., vestibular) system, and proprioception. The estimated body orientation is used by the CNS to send motor commands to the muscles to generate corrective muscle activity. This corrective muscle activity, together with intrinsic visco-elastic properties of the muscles and tendons, generate corrective joint torques to keep the body in an upright position (Peterka, 2003). This cycle of continuous sensory feedback of body orientation, corrective muscle activity, and corrective joint torques implies that balance control is effectively a closed-loop system.

In a closed-loop system it is difficult to separate cause and effect, as these are interrelated (Engelhart et al., 2014). For example, in balance control it is difficult to recognize how joint torques influence joint angles or vice versa. In order to “open” the loop, a unique independent input should be applied in the form of external perturbations (van der Kooij et al., 2005; Boonstra et al., 2013). The joint torque or body sway responses are correlated to the external perturbations to estimate the dynamic balance behavior. The dynamic balance behavior can be expressed in Frequency Response Functions describing the relation between perturbation and response in magnitude and relative timing (phase) for each excited frequency.

To estimate how each subsystem (i.e., sensory systems, CNS, muscles, and body) contributes to dynamic balance behavior, balance control models have been developed. Models are important tools to relate experimental data to physiologically relevant parameters that indicate the contribution of each subsystem to the dynamic behavior (Schouten et al., 2008; Mergner, 2010; Kiemel et al., 2011). Peterka proposed a simple and descriptive linear balance control model, the Independent Channel (IC) model, that consists of a single-link inverted pendulum, a neural controller with proportional, and derivative gains, a time delay and sensory feedback (Peterka, 2002) to adequately describe balance control in the face of sensory perturbations, like support surface rotations or visual surround rotations. Due to its simplicity, the model is frequently used and applied to obtain pathophysiological changes in certain (patient) groups, such as elderly (Pasma et al., 2015; Wiesmeier et al., 2015), vestibular loss patients (Peterka, 2002; Mergner et al., 2009; Peterka et al., 2011), and Parkinson's disease patients (Boonstra et al., 2014).

However, as balance control models contain multiple parameters and parameters interact, the relative contribution of each parameter to balance control is not directly evident (Engelhart et al., 2014). For example, reduced body sway could be due to both an increased stiffness of the system or to sensory reweighting (Peterka, 2002). To provide insight into the relative contribution of different balance control parameters, we adopted a method to investigate the sensitivity of individual model parameters to the overall dynamic balance behavior. The method is applied to the well-known and frequently used inverted pendulum balance control model of Peterka (2002) to get more insight in the sensitivity of the model parameters. We evaluated the sensitivity of each model parameter to determine how each parameter shapes the dynamic balance behavior (i.e., magnitude and phase as function of frequency). More insight in the unique contribution and relative importance of all parameters helps to understand the applicability and clinical utility of the model for identifying the important parameters that characterize balance control during stance, and to identify which parameters are crucial and critical to detect underlying changes in balance behavior with age and diseases.

## Materials and methods

**Figure 1:**
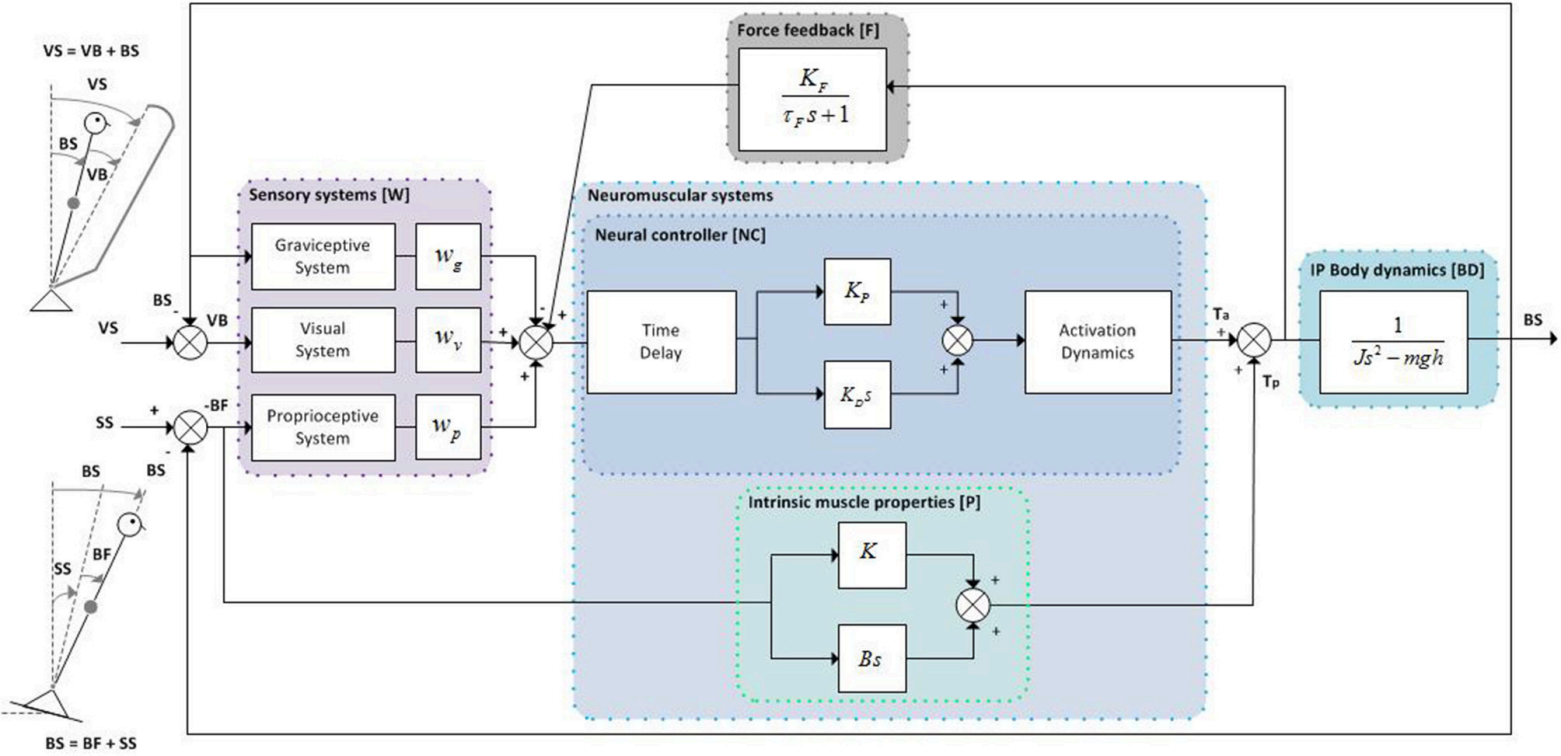
Inverted pendulum balance control model. The human body is represented by an inverted pendulum (IP) with corresponding body dynamics (*BD*), which is controlled by the neuromuscular system consisting of the neural controller (*NC*) incorporating a PD controller with neural signal transport delay and muscle activation dynamics, and the intrinsic muscle properties (*P*). The *NC* receives a weighted combination of body orientation information from the sensory systems (*W*) (i.e., graviceptive, visual, and proprioceptive system) and force feedback information (*F*) from the force sensors. The total corrective ankle torque consists of the output of the *NC* processed through muscle activation dynamics plus the torque arising from the intrinsic muscle properties (*P*). The proprioceptive and visual sensory systems can be perturbed by support surface (SS) and visual surround (VS) rotations, respectively.

The IC model (Peterka, 2002) comprises of sensory and motor components to describe dynamic balance behavior under external sensory perturbations (see Figure 1). The model parameters and the default values are presented in Table 1. The default values of the model parameters were based on the values found in experimental studies (van der Kooij and Peterka, 2011). Compared to Peterka (2002), activation dynamics were added to the model. As the activation dynamics interact with the time delay, a lower value for the time delay (0.097 s) was used compared with other studies, as found in previous studies which included the activation dynamics (van der Kooij and Peterka, 2011; Pasma et al., 2015). It should be noted that the neural controller integral gain (*K*_*I*_) was deleted from the model, since this gain was shown to be redundant in a model with a torque-related sensory channel, i.e., force feedback (Peterka, 2003), as also will be addressed in the Discussion section.

**Table 1:** Balance control parameters of the Independent Channel model with the chosen default values, based on experimental data.

**Symbol**	**Parameter**	**Default value**	
*J*	Moment of inertia [kg m^2^] (Fonteyn et al., 2010)	66	Based on anthropometry
*m*	Body mass [kg] (Fonteyn et al., 2010)	75	
*h*	Centre-of-mass height [m] (Fonteyn et al., 2010)	0.83	
*g*	Gravitational constant [m/s^2^]	9.81	Known
*K*	Intrinsic muscle stiffness [Nm/rad]	40.5	
*B*	Intrinsic muscle damping [Nms/rad]	68.8	
ω*_0_*	Activation dynamics—natural frequency [rad/s] (Mugge et al., 2010)	16.8	
β	Activation dynamics—relative damping [-] (Mugge et al., 2010)	0.99	
τ_*D*_	Time delay [s]	0.097	Model fitting
*W_*P*_*	Sensory proprioceptive weight [-]	0.8	
*W_*V*_*	Sensory visual weight [-]	0.8	
*K_*P*_*	Proportional gain [Nm/rad]	943.9	
*K_*D*_*	Derivative gain [Nms/rad]	313.5	
*K_*F*_*	Force feedback—gain [rad/Nm]	0.0018	
τ_*F*_	Force feedback—time constant [s]	17.4	

*The last column indicates how this parameter is typically obtained in experimental studies. The sensory weight depends on the perturbation, i.e., support surface or visual surround rotation, and represents the proprioceptive weight (W_P_), or visual weight (W_V_), respectively*.

### Model transfer functions

The resulting overall dynamic balance behavior was described with transfer functions (TFs) in the Laplace domain. The TFs provide a quantitative measure of how the perturbations affect the response (i.e., body sway) in regards of magnitude and relative timing (phase) as a function of frequency. The body sway responses to two common perturbations (i.e., support surface rotation and visual surround rotation) represented by the TFs from support surface rotation to body sway (*H*_*SS*_) and from visual surround rotation to body sway (*H*_*VS*_):

(1) $$H_{SS}\left(s\right)=\frac{BS\left(s\right)}{SS\left(s\right)}=\frac{P\;\cdot \;BS+W_{P}\;\cdot \;NC\;\cdot \;BD}{1-F\;\cdot \;NC+P\;\cdot \;BD+NC\;\cdot \;BD}\;$$

(2) $$H_{VS}\left(s\right)=\frac{BS\left(s\right)}{VS\left(s\right)}=\frac{W_{V}\;\cdot \;NC\;\cdot \;BD}{1-F\;\cdot \;NC+P\;\cdot \;BD+NC\;\cdot \;BD}$$

The individual model subsystems included in the TFs are described in more detail in Figure 1 and **Appendix A**. The model TFs were evaluated between 0.1 and 3 Hz, as these are the frequencies that dominate human balance behavior (Soames and Atha, 1982).

### Sensitivity analysis

A sensitivity analysis of the model parameters on the TFs was performed. A sensitivity analysis indicates how uncertainty in the TFs can be apportioned to different sources of uncertainty in the model parameters (Saltelli et al., 2008). We applied a widely used approach of sensitivity analysis, namely “local” sensitivity analysis, which generally is derivative based (numerical or analytical). Local sensitivity analysis depends on the operating point (Saltelli et al., 2008) and only investigates the impact on the TFs based on changes only very close to the nominal values. As patients may be at a different operating point, we investigated the effect of changing the default values, and therefore changing the operating point, on the local sensitivity.

#### Local sensitivity

Local sensitivity analysis is a “one-at-a-time” technique, as only the effect of a single parameter of the system is analyzed at a time, while the rest of the parameters remain fixed at the chosen value (i.e., the operating point). The sensitivity of each parameter (*p*) of the balance control model was determined by taking the partial derivative of both TFs (see Equations 1 and 2) using the Matlab symbolic toolbox (Version 2014b, Mathworks, Natick, MA):

(3) $$S_{i}\left(p,f\right)=\frac{\partial H\left(p,f\right)}{\partial p_{i}}$$

where *S*_*i*_ is the sensitivity of parameter *p*_*i*_ for a specific parameter set *p* and frequency range *f*, and $\frac{\partial H\left(p,f\right)}{\partial p_{i}}$ the partial derivative of the TF *H*.

The sensitivity is a function of frequency consisting of complex numbers, as the TF is also a function of frequency with complex numbers. Therefore, it was investigated in both the magnitude and phase of the TF which frequency region each model parameter has its largest effect on the dynamic balance behavior, i.e., for which frequencies the partial derivative is the largest.

To show the unique contribution of the parameters per frequency, we multiplied the partial derivatives with a scaling factor (*SF*) such that for each parameter the relative maximal change of the TF magnitude equals 0.7 (an arbitrary number; Equation 4).

(4) $$SF_{i}=\frac{0.7}{max\left(\frac{{\text{comp}}_{H}S_{i}\left(p,f\right)}{\left|H\left(p,f\right)\right|}\right)}$$

where *SF*_*i*_ is the scaling factor of parameter *p*_*i*_, comp_*H*_*S*_*i*_(*p, f*) represents the component of *S*_*i*_(*p,f*) with respect to TF *H* of the sensitivity of parameter *p*_*i*_ for a specific parameter set *p* and frequency range *f* and |*H(p,f)|* the magnitude of TF *H*.

**Figure 2:**
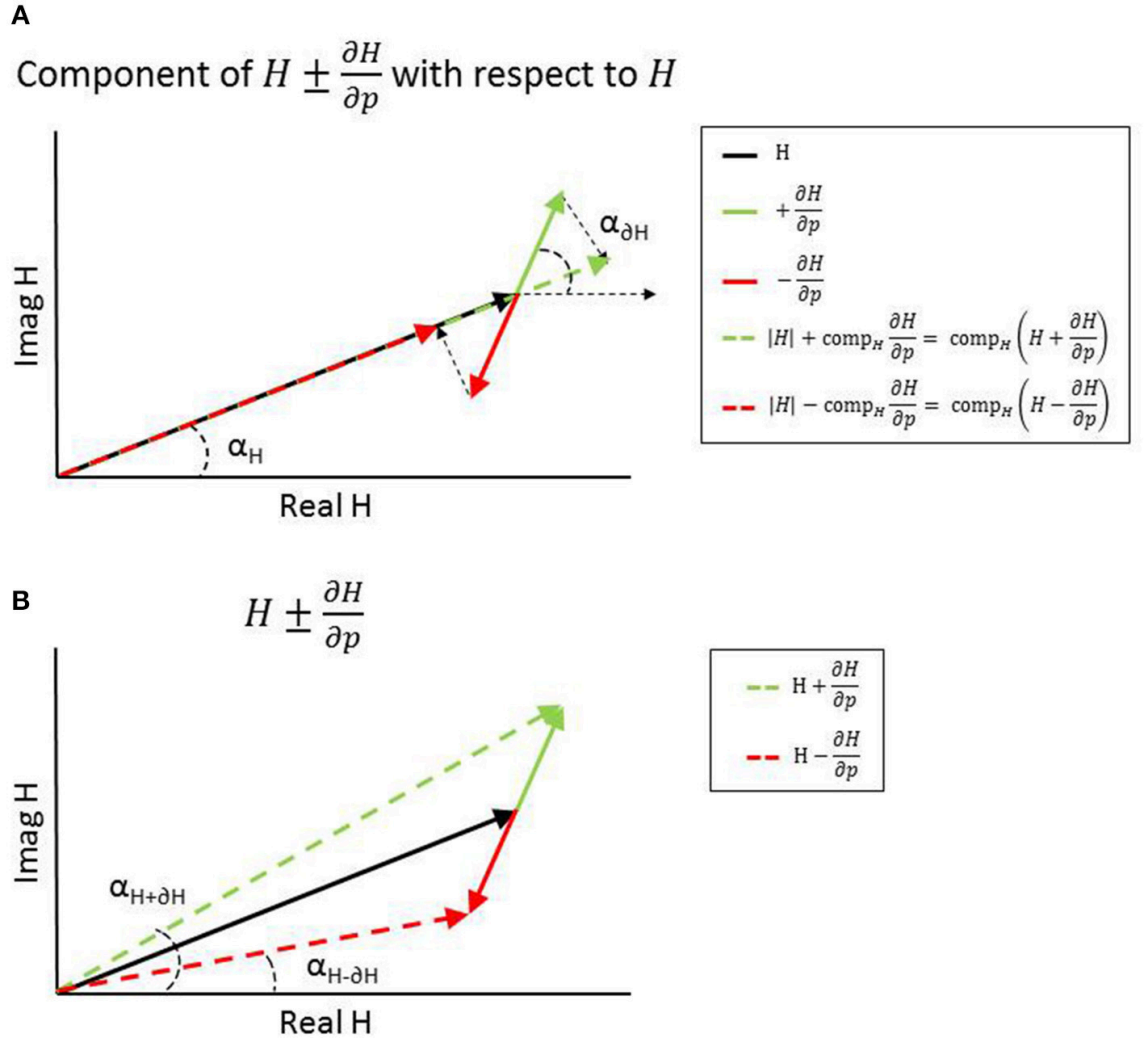
Representation of adding (green) or subtracting (red) the (normalized or scaled) partial derivatives from the transfer function *H* at one frequency resulting in a change in magnitude **(A)** and change in phase **(B)**.

This scaling factor was used in Equation (5) to calculate the resulting transfer function by adding or subtracting the scaled sensitivity.

(5) $$\begin{array}{l} H^{+}\left(p,f\right)=H\left(p,f\right)+SF_{i}\;\cdot \;S_{i}\left(p,f\right)\\ \;H^{-}\left(p,f\right)=H\left(p,f\right)-SF_{i}\;\cdot \;S_{i}\left(p,f\right) \end{array}$$

where *S*_*i*_ is the sensitivity of parameter *p*_*i*_ for a specific parameter set *p* and frequency range *f* and *SF*_*i*_ the scaling factor of parameter *p*_*i*_. The component of *H*^+^(*p,f*) and *H*^−^(*p,f*) with respect to TF *H* represents the magnitude of *H*^+^(*p,f*) and *H*^−^(*p,f*), while the angle of *H*^+^(*p,f*) and *H*^−^(*p,f*) represents the phase angle of *H*^+^(*p,f*) and *H*^−^(*p,f*) (see Figure 2).

To compare the sensitivity of each parameter, a normalized sensitivity was calculated, as the sensitivity depends on the units of the parameters and the transfer function, for which the changes were expressed as proportions (Norton, 2015; Equation 6). The normalized partial derivatives are comparable across parameters despite the different units of the parameters.

(6) $$S_{n,i}\left(p,f\right)=\frac{p_{i}}{\left|H\left(p,f\right)\right|}S_{i}\left(p,f\right)$$

where *S*_*i*_ is the sensitivity of parameter *p*_*i*_ for a specific parameter set *p* and frequency range *f* and |*H*(*p, f*)| the magnitude of TF *H*. The normalized partial derivatives are expressed by a magnitude, representing the component of *S*_*n,i*_ with respect to *H*, and a phase angle representing the angle difference between *H* plus *S*_*n,i*_ and *H* (see Figure 2).

To indicate the relative importance of each parameter, an importance measure (*IMP*) was calculated (Equation 7).

(7) $$IMP_{i}=\int_{0.1}^{3}{\text{comp}}_{H}S_{n,i}\left(p,f\right)\;d\;\left(\log\left(f\right)\right)$$

where comp_*H*_*S*_*n,i*_ is the component of the normalized sensitivity of parameter *p*_*i*_ for a specific parameter set *p* and frequency range *f* with respect to TF *H*. A high value indicates a high parameter sensitivity; a small change in the parameter value results in a large change in the TF magnitude.

#### Operating points

Local sensitivity analysis estimates the partial derivatives at a particular point in the model parameter space. However, it is likely that the model parameters varied within a range from person to person due to age and disease. The effect of the model parameters on the balance behavior is nonlinear, which results in a change of the sensitivity at different operating points of the model. Therefore, we investigated the effect on the partial derivatives of all parameters by systematically changing parameters.

Previous studies showed that the time delay and the sensory weights are important parameters changing with age and diseases (Peterka, 2002; Cenciarini et al., 2010; Pasma et al., 2015; Wiesmeier et al., 2015; Engelhart et al., 2016). To investigate the effect of these parameter changes on the sensitivity of all parameters on the TF, we changed the time delay and sensory weight systematically from −30% till +30%, resulting in seven partial derivatives per parameter and investigated the effect on the normalized partial derivatives of all parameters.

## Results

### Sensitivity of balance control parameters: from support surface rotation to body sway

**Figure 3:**
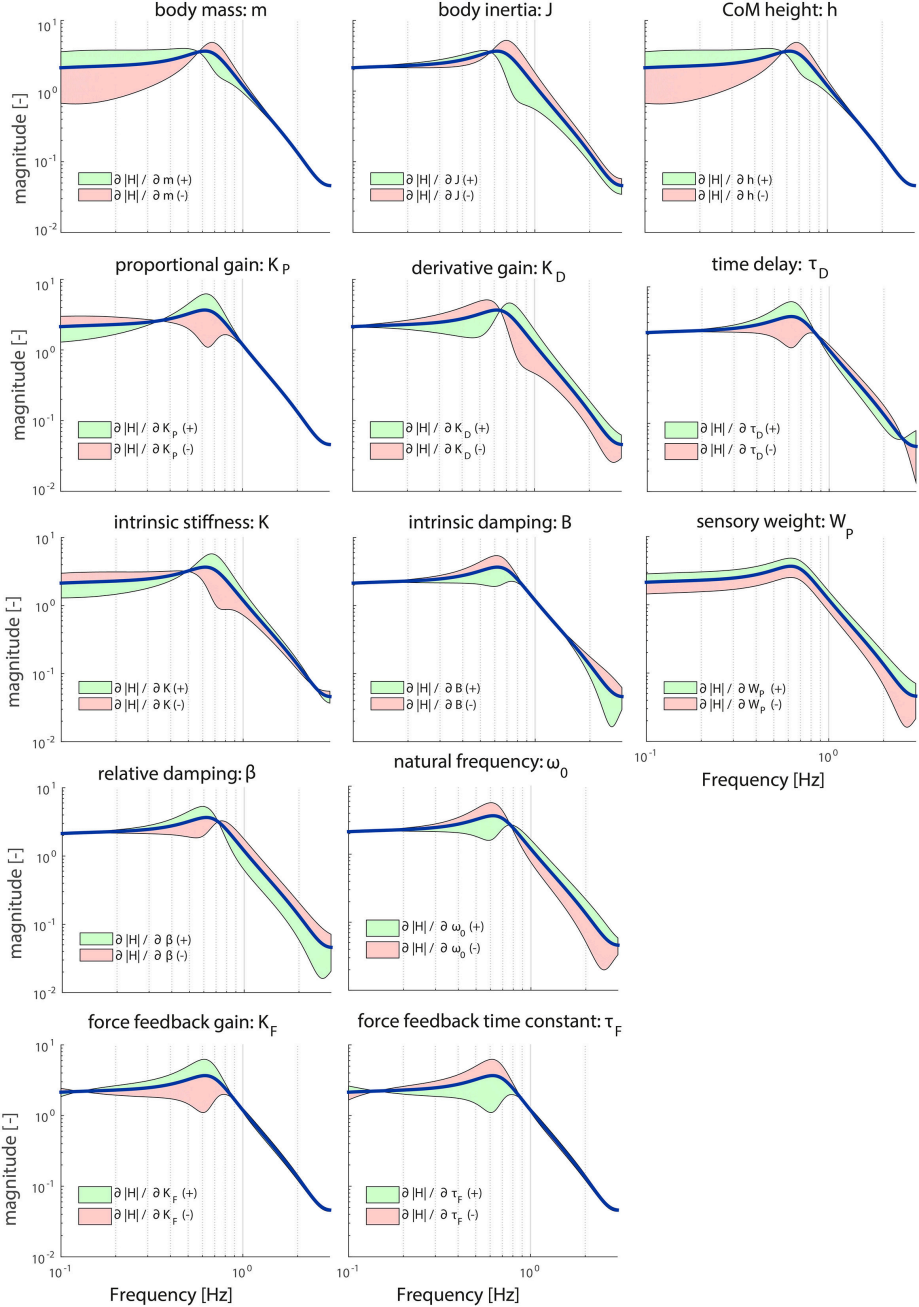
Sensitivity of each parameter of the model's transfer function from support surface rotation to body sway (*H*_*SS*_) on the TF magnitude represented by adding or subtracting the partial derivatives from the TF magnitude; green indicates the effect of a parameter increase and red a parameter decrease. The partial derivatives are multiplied by a scaling factor such that the relative maximum change of the magnitude is 0.7.

**Figure 4:**
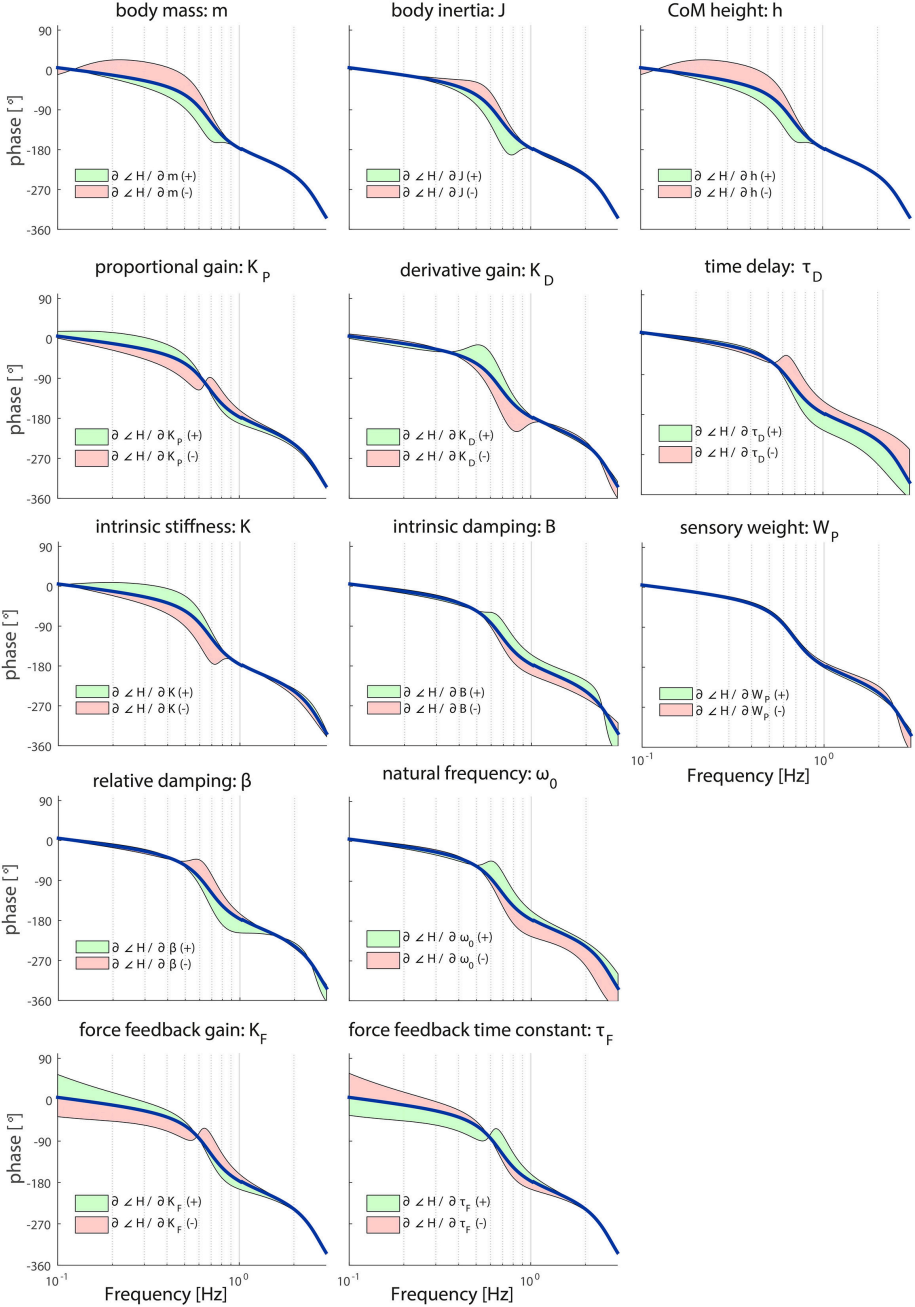
Sensitivity of each parameter of the model's transfer function from support surface rotation to body sway (*H*_*SS*_) on the TF phase represented by adding or subtracting the partial derivatives from the TF phase; green indicates the effect of a parameter increase and red a parameter decrease. The partial derivatives are multiplied by a scaling factor such that the relative maximum change of the magnitude is 0.7.

Figures 3, 4 illustrate the sensitivity of the model parameters on the TF magnitude and phase, respectively, in the chosen operating point reported in Table 1. The anthropometric parameters body mass (*m*) and Center of Mass height (*h*) influence the shape of the TF magnitude between 0.1 and 1 Hz in a similar fashion; between 0.1 and 0.6 Hz increasing these parameters leads to a larger magnitude, while decreasing these parameters leads to a smaller magnitude. At frequencies between 0.6 and 1 Hz, this effect is reversed. Both parameters influence the TF magnitude mostly on the low frequencies. The moment of inertia (*J*) influences the shape of the TF magnitude mostly above 0.6 Hz; increasing its value decreases the magnitude and vice versa. In other words, a decrease in *m, h*, and *J* effectually increases the resonance peak of the magnitude.

The proportional gain (*K*_*P*_) shapes the TF magnitude mainly in the lower frequencies (0.1–1 Hz); increasing *K*_*P*_ makes the resonance peak of the magnitude more pronounced. *K*_*P*_ mostly influences the resonance peak. The derivative gain (*K*_*D*_) shapes the magnitude over the whole frequency range; increasing *K*_*D*_ results in a lower magnitude at the lower frequencies (0.1–0.6 Hz) and a higher magnitude at the higher frequencies (0.6–3 Hz).

The intrinsic stiffness (*K*) has a similar effect as *K*_*P*_. However, *K* shapes the TF magnitude over the whole frequency range, whereas *K*_*P*_ influences the TF magnitude until 1 Hz. Intrinsic damping (*B*) mainly affects the resonance peak of the magnitude between 0.3 and 0.8 Hz.

Both muscle activation parameters (ω_0_ and β), as well as the force feedback parameters (*K*_*F*_ and τ_*F*_) shape the TF magnitude in a similar fashion. ω_0_ and τ_*F*_ influence the shape of the magnitude in reverse directions compared with β and *K*_*F*_, leading to an increase of the resonance peak of the magnitude with decreasing ω_0_ and τ_*F*_ and increasing β and *K*_*F*_. In contrast with the force feedback parameters, the muscle activation parameters also influence the higher frequencies.

The sensory weight (*W*_*P*_) has a pronounced effect on the whole frequency range of the TF magnitude; increasing *W*_*P*_ results in an increase of the TF magnitude on all frequencies. The time delay (τ_*D*_) predominantly affects the frequencies around the resonance peak (i.e., 0.6 Hz); increasing τ_*D*_ increases the resonance peak at this frequency point, which is in accordance with the destabilizing effect of a time delay in a feedback loop.

As shown in Figure 4, the shape of the TF phase is influenced between 0.1 and 1 Hz in a similar fashion by the anthropometric parameters *m* and *h*; increasing these parameters leads to an increased phase lag between 0.1 and 1 Hz and vice versa. *J* has effect on the TF phase at the frequencies around the resonance peak (i.e., 0.6 Hz); increasing *J* leads to an increased phase lag and vice versa.

*K*_*D*_ has also an effect on the TF phase at the frequencies around the resonance peak (i.e., 0.6 Hz); increasing *K*_*D*_ leads to a decreased phase lag between 0.3 and 1 Hz and vice versa. *K*_*P*_ and *K* have a similar effect on the phase; increasing these parameters leads to a decreased phase lag between 0.1 and 0.6 Hz and vice versa. However, the effect of *K*_*P*_ reverses at between 0.6 and 0.7 Hz, whereas the effect of *K* is equal till 0.8 Hz.

*B*, ω_0_ and β all influence the TF phase between 0.5 and 3 Hz. Increasing ω_0_ and *B* decreases the phase lag, whereas decreasing ω_0_ and *B* has the opposite effects. For β these effects are reversed compared with ω_0_ and *B*. The force feedback parameters *K*_*F*_ and τ_*F*_ have opposite effects on the TF phase; increasing *K*_*F*_ leads to a decreased phase lag and vice versa at lower frequencies. This effect is reversed above 0.6 Hz. In contrast with the muscle activation parameters, the force feedback parameters also influences the TF phase on the low frequencies.

*W*_*P*_ hardly affects the shape of the TF phase. In contrast, τ_*D*_ clearly influences the TF phase. The dominant effect can be observed at the frequencies around the resonance peak (i.e., 0.6 Hz); increasing τ_*D*_ becomes apparent for the TF phase above these frequencies resulting in an increased phase lag.

Table 2 presents the importance measures of each parameter. The importance measures of *K, B, K*_*F*_, and τ_*F*_ are low (<0.5) compared with the other parameters. This means that these parameters have to change more to get a comparable change as the other parameters. *J, W*_*P*_, and *K*_*D*_ show the highest important measures; the TF magnitude is most sensitive for changes in these parameters.

**Table 2:** Relative importance of parameters.

**Parameter**	***H_*SS*_***	***H_*VS*_***
Moment of inertia (*J*)	1.30	1.30
Body mass (*m*)	1.19	1.19
Centre-of-mass height (*h*)	1.19	1.19
Intrinsic muscle stiffness (*K*)	0.06	0.08
Intrinsic muscle damping (*B*)	0.39	0.26
Activation dynamics—natural frequency (ω*_0_*)	1.20	0.99
Activation dynamics—relative damping (β)	1.26	1.04
Time delay (τ_*D*_)	0.75	0.59
Sensory proprioceptive weight (*W_*P*_*)	1.58	–
Sensory visual weight (*W_*V*_*)	–	1.48
Proportional gain (*K_*P*_*)	1.19	1.18
Derivative gain (*K_*D*_*)	1.88	1.70
Force feedback—gain (*K_*F*_*)	0.08	0.08
Force feedback—time constant (τ_*F*_)	0.08	0.08

*The importance measure represents the relative importance of each parameter. Importance measures are presented for both TFs, namely H_SS_ and H_VS_*.

### Sensitivity of balance control parameters: from visual surround rotation to body sway

**Figure 5:**
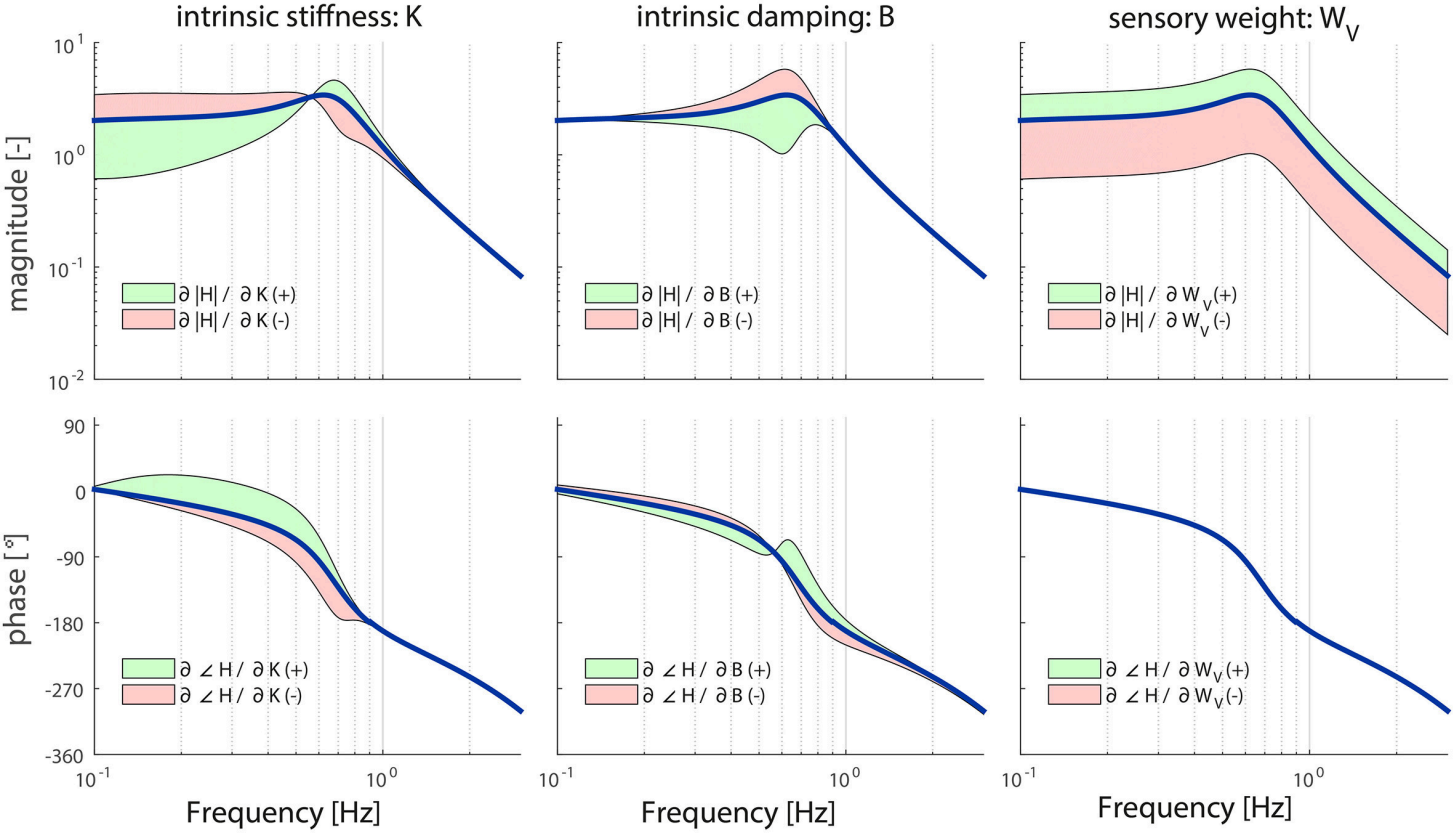
Sensitivity of the sensory weight (*W*_*V*_), intrinsic stiffness (*K*), and intrinsic damping (*B*) of the model's transfer function from visual surround rotation to body sway (*H*_*VS*_) on the magnitude and phase represented by adding or subtracting the partial derivatives from the TF magnitude or phase; green indicates the effect of a parameter increase and red a parameter decrease. The partial derivatives are multiplied by a scaling factor such that the relative maximum change of the magnitude is 0.7.

For almost all parameters, the results of the local sensitivity analysis of the TF from visual surround rotation to body sway are comparable to the sensitivity analysis of the TF from support surface rotation to body sway. However, the parameters *K* and *B* did influence the TF in a different way as shown in Figure 5. Increasing or decreasing *K* has a more pronounced effect on *H*_*VS*_ in the lower frequency ranges, compared to *H*_*SS*_; increasing *K* leads to larger decreases in *H*_*VS*_ compared to *H*_*SS*_ for frequencies up to 0.6 Hz. Contrary, *B* has a less pronounced effect on the higher frequencies (>2 Hz) of the *H*_*VS*_ compared to the *H*_*SS*_: increases in *B* do not have an effect on the *H*_*VS*_ compared for frequencies larger than 2 Hz, whereas *B* did influence *H*_*SS*_ in this region.

The sensory weight *W*_*V*_ influences the TF magnitude on all frequencies, which is comparable to *W*_*P*_. However, the effect is equal over all frequencies. In contrast with *W*_*P*_, *W*_*V*_ does not influence the TF phase.

The importance measures (Table 2) are comparable with the importance measures as found in the sensitivity analysis of TF from support surface rotation to body sway. The importance measures of most of the parameters are clearly lower, indicating that *H*_*VS*_ is less sensitive to changes of most of the parameters compared with *H*_*SS*_. Furthermore, *H*_*VS*_ is sensitive to changes in *W*_*V*_, but less sensitive compared to *H*_*SS*_ to changes in *W*_*P*_.

### Effect of operating point

**Figure 6:**
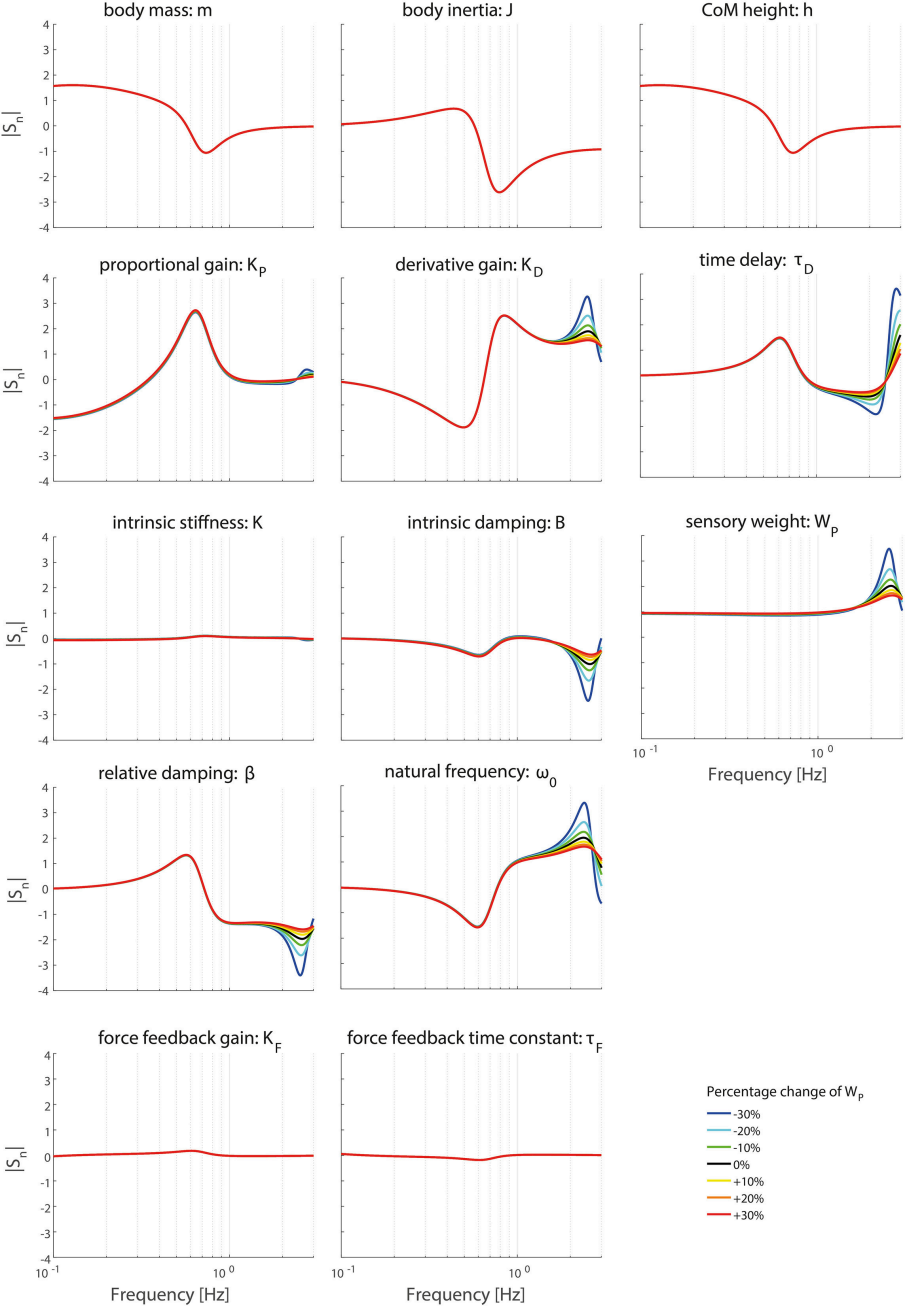
Change in the normalized sensitivity of each parameter of the model's transfer function from support surface rotation to body sway (*H*_*SS*_) on the TF magnitude with systematically changing the sensory weight.

**Figure 7:**
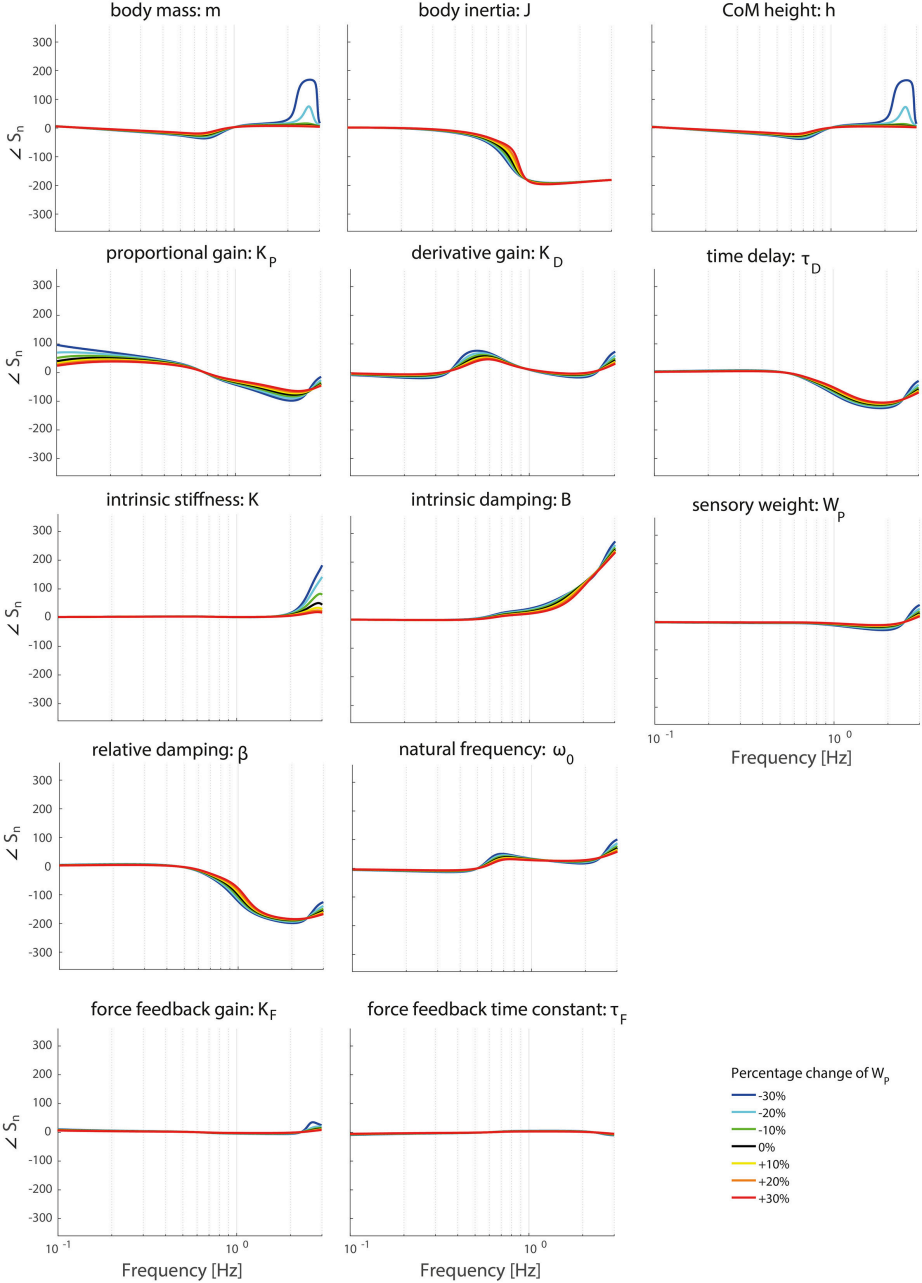
Change in the normalized sensitivity of each parameter of the model's transfer function from support surface rotation to body sway (*H*_*SS*_) on the TF phase with systematically changing the sensory weight.

**Figure 8:**
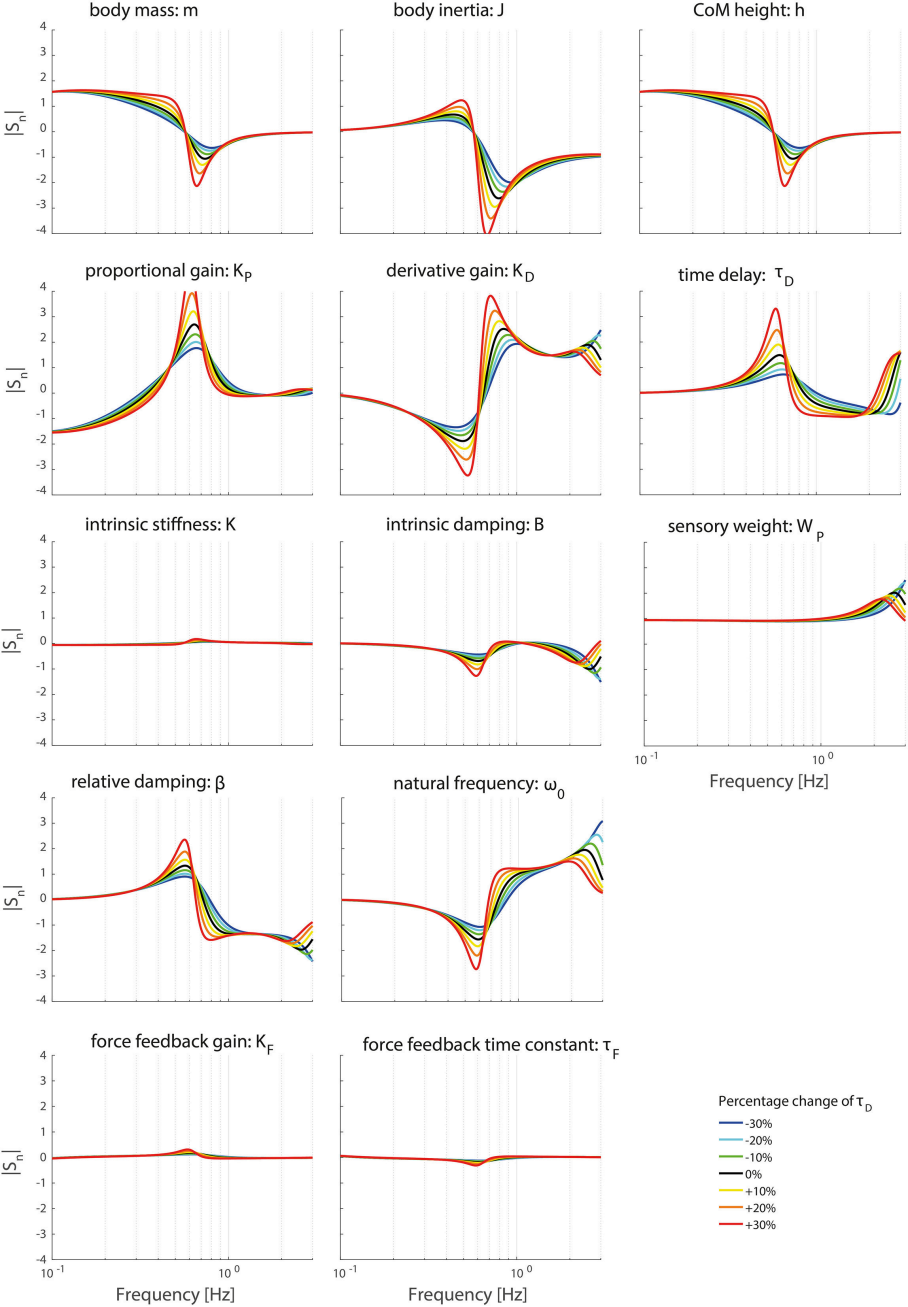
Change in the normalized sensitivity of each parameter of the model's transfer function from support surface rotation to body sway (*H*_*SS*_) on the TF magnitude with systematically changing the time delay.

**Figure 9:**
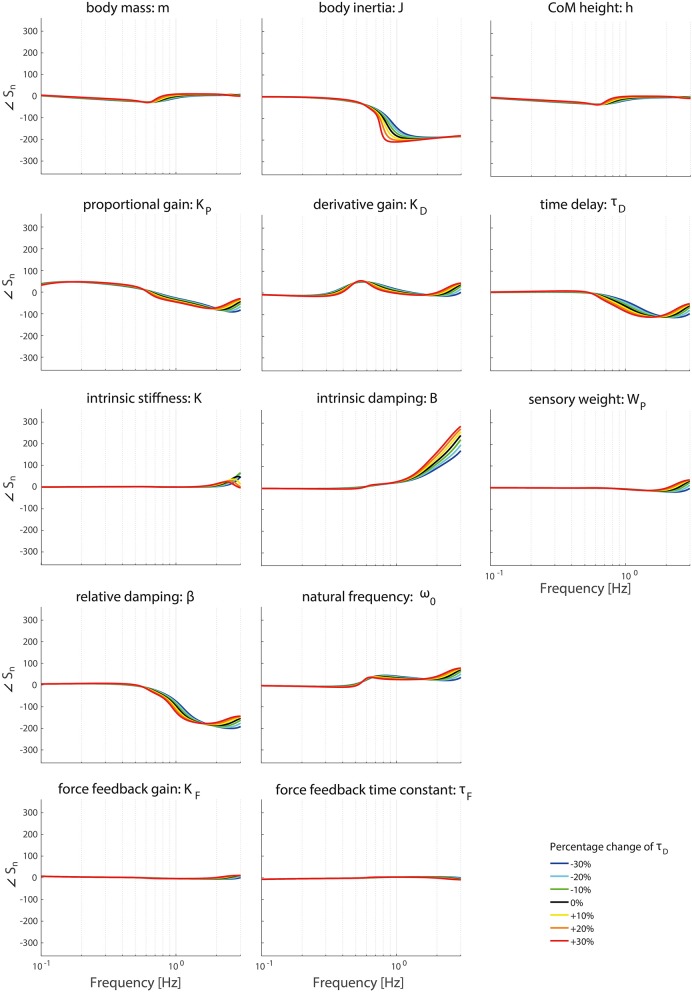
Change in the normalized sensitivity of each parameter of the model's transfer function from support surface rotation to body sway (*H*_*SS*_) on the TF phase with systematically changing the time delay.

Figures 6–**9** show the effect of systematically changing the sensory weight and time delay on the normalized sensitivity of all parameters. Figures 6, 7 clearly show that changing the sensory weight mainly effects the sensitivity of *K*_*P*_, *K*_*D*_, *B*, ω_0_, β, τ_*D*_, and *W*_*P*_ on the TF magnitude on the high frequencies. A higher *W*_*P*_ results in less sensitivity on the high frequencies. Also, the sensitivity on TF phase is mainly influenced on high frequencies.

Figures 8, 9 clearly show the influence of changing the time delay on the normalized sensitivity of all parameters; increasing the time delay results in a higher sensitivity of all parameters around the resonance peak for both the TF magnitude and TF phase. Furthermore, the frequency of the highest sensitivity changes.

## Discussion

In this study we performed a local sensitivity analysis based on partial derivatives of a commonly used balance control model (i.e., the IC model) to investigate the unique contribution and the relative importance of the parameters incorporated in the model. The results showed that all parameters shape the TF magnitude and phase and therefore play a role in the balance behavior. However, the importance of the parameters varies. The sensory weight, the time delay, the derivative gain, and the proportional gain are of relatively high importance and show a unique contribution to the TF, suggesting that the used balance control model is suitable for the identification of these parameters with experimental data, which is confirmed by other studies showing changes in these parameters with age and disease (Peterka, 2002; Cenciarini et al., 2010; Pasma et al., 2015; Wiesmeier et al., 2015).

### Sensitivity analysis of a balance control model

Balance control models consist of several parameters which could interact. Therefore, it is difficult to recognize the unique contribution and relative importance of each parameter to balance control. The results of the sensitivity analysis help with optimizing the balance control models and show the unique contribution of each parameter. In case parameters have similar contributions to the TF, this indicates that these parameters interact and therefore are difficult to distinguish, such as the intrinsic stiffness and proportional gain. The model can be simplified by eliminating some of these parameters or by giving some parameters a constant value obtained from different experiments.

The sensitivity analysis also shows the relative importance of each parameter represented by the importance measures. The TF is less sensitive to changes in parameters with low importance measures, which indicates that these parameters are less important to describe changes in dynamic balance behavior, such as the force feedback parameters and the intrinsic stiffness. Therefore, these parameters can be kept constant or can even be removed from the model. However, this might influence the sensitivity of the other parameters by changing the operating point. On the other hand, parameters with a high importance measure indicate high sensitivity and it is recommended to focus on these parameters.

### Many parameters influence the transfer function in a similar fashion

The results showed that many parameters act in the same frequency range and shape the TF in a similar fashion. For example, both the proportional gain (*K*_*P*_) and intrinsic stiffness (*K*) similarly shape the magnitude of the TF in the lower frequencies (0.1–1 Hz). This is a very well-known effect, which makes it very difficult to disentangle the effect of the intrinsic and reflexive (i.e., proportional gain) stiffness. However, for upright stance, it has been shown many times that the contribution of the intrinsic stiffness is low (i.e., about 10%), compared to the reflexive stiffness (Peterka, 2002; Vlutters et al., 2015). This is also shown by the low importance measure for the intrinsic stiffness compared with the importance measure of the proportional gain. Therefore, lumping these two parameters would not give rise to large errors in estimations of ankle stiffness and in descriptions of the dynamic balance behavior. However, as consequence no distinction could be made between the intrinsic stiffness and proportional gain. Therefore, possible differences in intrinsic stiffness and proportional gain between patient groups would not be detectable.

The force feedback (*K*_*F*_, τ_*F*_) is effective in the low and mid frequencies (0.2–0.8 Hz), with an inverse relationship between the influence of each of the two parameters on the TF. This indicates that there is some interaction between the parameters and overparameterization, which can be solved by combining those parameters into one parameter or removing one of the parameters. As mentioned previously, the integral gain (*K*_*I*_) and the force feedback gain also have similar influence on the TF at low frequencies as shown by previous studies (Peterka, 2003). This can also be shown with a sensitivity analysis to support the decision to remove *K*_*I*_ from the model, which will nicely illustrate the practical use of sensitivity analysis.

The sensory weight (*W*_*P*_) influenced the magnitude of the TF across the whole frequency range; an increase in *W*_*P*_ increased the magnitude and vice versa, while the phase of the TF remained unaffected. This confirms previous studies, which showed that when participants increase their sensory weighting, this can be easily extracted from the TF, by searching for increases or decreases in the magnitude of the TF over the whole frequency range (Peterka, 2002; Pasma et al., 2012). However, the effect of the other parameters on the TF still needs to be taken into account.

On the other hand, the visual weight *W*_*V*_ and the proprioceptive weight *W*_*P*_ have a different effect on the TFs. This can be explained by Equations (1, 2). With support surface rotations not only the proprioceptive weight, but also the intrinsic dynamics interact. This is represented by the inclusion of both the intrinsic dynamics and sensory weight factors in the numerator of Equation 1. Therefore, *W*_*P*_ does not act as a constant gain like *W*_*V*_ does.

### Influence of operating point

It should be noted though that the results of sensitivity analysis vary with the operating point chosen. The operating point (i.e., the set of values for model parameters) chosen in this study (Table 1) is obtained from studies in healthy subjects (Peterka, 2002; van der Kooij and Peterka, 2011). Therefore, it remains to be elucidated whether different values of model parameters in subjects with altered neuromuscular dynamics (e.g., elderly) affect the TF in a similar way as in healthy young subjects, as it is reasonable to assume that they have a different operating point, due to e.g., increased proprioceptive weight (Pasma et al., 2015; Wiesmeier et al., 2015) or time delay (Engelhart et al., 2016) or increased derivative and proportional gain (Cenciarini et al., 2010).

Changing the operating point with respect to the sensory weight only lowers the sensitivity in the higher frequency regions of both the TF magnitude and TF phase, i.e., a lower importance measure, with increasing proprioceptive weight. Changing the operating point with respect to the sensory weight does not influence the frequency regions the parameters act on.

Changing the operating point with respect to the time delay does influence the frequency regions the parameters act on. The frequency of the maximal partial derivative changes, i.e., the resonance peak changes. Furthermore, the maximal change itself increases with increasing time delay, representing a higher sensitivity and therefore a higher importance measure. In other words, the TFs become more sensitive to changes of all parameters in case of a higher time delay.

Future studies that combine sensitivity analysis and experimental research are needed to gain insight into the role of altered neuromuscular dynamics or reweighting strategies on parameter estimates of balance control models.

### Practical applications

Sensitivity analysis can be used to predict how changes in model parameters, induced by pathophysiological conditions, may affect balance control. Based on existing knowledge about pathophysiological changes in patient groups, sensitivity analysis can be used to formulate explicit hypotheses with respect to changes in the TF that these pathophysiological mechanisms induce. For example, as we know that elderly with polyneuropathy have less reliable proprioceptive information, this might result in less use of the proprioceptive information, i.e., a decrease in the sensory weight of proprioceptive information *W*_*P*_. Therefore, we can expect a decrease of the TF over the whole frequency range. Furthermore, we can hypothesize an increase of sensitivity of the other parameters on the high frequencies. Hence, sensitivity analyses in experimental data may lead to more hypothesis-driven instead of explorative research and ultimately to a better interpretation for observed changes in balance control in people with balance disorders. However, one always has to keep in mind that several parameters could have the same effect on the TF.

Sensitivity analysis also might play an important role in designing new experiments and perturbation signals. From the sensitivity analysis, it is possible to gain more insight in which regions of the TF are influenced by which parameter. Based on the research question and hypothesis, one can identify the parameters of interest and therefore the regions of interest, i.e., the frequencies which are influenced by the parameters of interest. By designing a perturbation signal with more power on the frequencies of interest, the value of the corresponding parameters might be estimated more reliably.

### Limitations

In the current study we used the IC model to describe balance control, which is a linearized and simplified balance control model and therefore frequently used in literature to describe balance control in healthy and clinical populations. The used model and methods assume that the behavior of the participants is linear and does not change over time. Furthermore, it assumes that the body moves as a single inverted pendulum and is stabilized primarily by the ankle torques. These assumptions are justified during small movements.

However, the IC model cannot be used in case of large movements and to investigate (a) adaptive behavior, (b) multi-segmental balance control, and (c) cognitive effects, as the model might miss some essential details. In that case, other models might be more interesting to use to describe balance control, like nonlinear models, intermittent control models, PDA control models, and multi-segmental models (Mergner, 2007; Insperger et al., 2013; Gawthrop et al., 2014; Hwang et al., 2016). The sensitivity analysis used in this study may also applicable to these kind of models and will provide more insight in the relative importance and contribution of model parameters and the applicability of the models.

## Conclusion

The local sensitivity analysis performed in this study contributes to the better understanding of the balance control model. It gives more insight in the unique contribution of all parameters on the shape of the TF and shows the relative importance of all parameters. The force feedback parameters and the intrinsic parameters are relatively less important, while the sensory weight, the time delay, the derivative gain, and the proportional gain are more important and uniquely contribute to the TF. This suggests that the used balance control model is suitable for the identification of the sensory weight, time delay and reflexive dynamics with experimental data, which potentially makes it able to identify differences between individuals and among different patient groups. The comparison of reliably identified parameters can give insight into the mechanisms that contribute to changes with age and with disease processes.

## Author contributions

All authors contributed substantially to this study; all contributed to the conception and design of the study, and interpretation of the results. TB and JP performed the analysis. JP and TB drafted the manuscript. JvK, VV, and AS revised the manuscript critically. All authors gave final approval of the submission.

### Conflict of interest statement

The authors declare that the research was conducted in the absence of any commercial or financial relationships that could be construed as a potential conflict of interest.

## Appendix A-human balance control model

In the model, the human body is represented by an inverted pendulum rotating about the ankle joint. Input of the system is the support surface or visual surround rotations and the output is the body sway (see Figure 1). The derivation of the model subsystems is described below.

### Human body dynamics

The human body dynamics (*BD*) are described by a single link inverted pendulum, incorporating the ankle joint, as in:

(A1) $$BD\left(s\right)=\frac{1}{Js^{2}-mgh}$$

where *J* is the body's moment of inertia about the ankle joint axis and *m* the body mass; *g* is the gravitational constant and *h* is the height of the Center of Mass above the ankle joint, and *s* is the Laplace variable.

### Sensory systems

The visual, proprioceptive and graviceptive (i.e., vestibular) sensory systems provide information regarding body orientation with respect to their individual internal reference. In this Independent Channel (IC) model (Peterka, 2002) the contribution of the sensory systems can be represented by relative weights, with one weight per sensory channel (i.e., *W*_V_, *W*_P_, *W*_G_). The sensory weights are summed up to 1, providing all the sensory information required to detect body orientation in space and estimate deviations from an upright posture (Cenciarini and Peterka, 2006).

### Force feedback

A torque-related sensory channel is included in the model to better explain low-frequency dynamics (Peterka, 2003). This positive force feedback (*F*), which is added to the visual, proprioceptive and graviceptive sensory signals, can be described by:

(A2) $$F\left(s\right)=\frac{K_{F}}{\tau_{F}s+1}$$

where *K*_F_ and τ_F_ are the force feedback's gain and time constant, respectively.

### Neuromuscular system

The neuromuscular system processes the motion- and force-related feedback to generate corrective torques in the ankle joint and stabilize posture. The neuromuscular system consists of two main components; the intrinsic and reflexive dynamics.

#### Intrinsic dynamics

The intrinsic dynamics (*P*) describe the tonic muscle activation, related to the intrinsic properties of muscles and tendons, i.e., visco-elasticity, and act without a time delay, containing a stiffness (*K*) and a damping component (*B*) as in:

(A3) P(s)=K+Bs

#### Reflexive dynamics

The reflexive dynamics represent the reflexive muscle activation, i.e., feedback control of the system, and comprise the neural controller along with the (muscle) activation dynamics. The neural controller, by incorporating proportional and derivative components (i.e., PD control), processes the afferent sensory signals, and subsequently generates appropriate motor commands to activate the muscle systems. The reflexive feedback acts with a time delay, due to neural signal processing and transduction. The total transfer function of the reflexive dynamics (*NC*), combining the neural signal transport delay with the PD controller and the muscle activation dynamics is the following.

(A4) $$NC\left(s\right)=e^{\tau_{D}s}\;\cdot \;\left(K_{P}+K_{D}s\right)\;\cdot \;\frac{{\omega_{0}}^{2}}{s^{2}+s\beta {\omega_{0}}^{2}+{\omega_{0}}^{2}}$$

where τ_D_ is the time delay; *K*_P_ and *K*_D_ are the controller's proportional and derivative gains resp.; ω_0_ and β are the natural frequency and the relative damping of the muscle activation dynamics.
